# Depression and health behaviors in Brazilian adults – PNS 2013

**DOI:** 10.1590/S1518-8787.2017051000084

**Published:** 2017-06-01

**Authors:** Marilisa Berti de Azevedo Barros, Margareth Guimarães Lima, Renata Cruz Soares de Azevedo, Lhais Barbosa de Paula Medina, Claudia de Souza Lopes, Paulo Rossi Menezes, Deborah Carvalho Malta

**Affiliations:** I Departamento de Saúde Coletiva. Faculdade de Ciências Médicas. Universidade Estadual de Campinas. Campinas, SP, Brasil; II Departamento de Psicologia Médica e Psiquiatria. Faculdade de Ciências Médicas. Universidade Estadual de Campinas. Campinas, SP, Brasil; III Programa de Pós-Graduação em Saúde Coletiva. Universidade Estadual de Campinas. Campinas, SP, Brasil; IV Departamento de Epidemiologia. Instituto de Medicina Social. Universidade do Estado do Rio de Janeiro. Rio de Janeiro, RJ, Brasil; V Departamento de Medicina Preventiva. Faculdade de Medicina. Universidade de São Paulo. São Paulo, SP, Brasil; VI Departamento de Enfermagem Materno Infantil e Saúde Pública. Escola de Enfermagem. Universidade Federal de Minas Gerais. Belo Horizonte, MG, Brasil

**Keywords:** Depressive Disorder, epidemiology, Health Behavior, Health Knowledge, Attitudes, Practice, Health Surveys, Transtorno Depressivo, epidemiologia, Comportamentos Saudáveis, Conhecimentos, Atitudes e Prática em Saúde, Inquéritos Epidemiológicos

## Abstract

**OBJECTIVE:**

To evaluate the prevalence of health-related behaviors according to presence and type of depression in Brazilian adults.

**METHODS:**

Based on a sample of 49,025 adults (18 to 59 years) from the National Survey on Health 2013 (PNS 2013), we estimated the prevalence of health-related behaviors (smoking; passive smoking; frequent or risky alcohol consumption; leisure time physical activity; time watching TV; and eating pattern indicators), according to the presence of depression (minor and major), evaluated by the Patient Health Questionnaire – 9 (PHQ-9), and the report of depressive mood (in up to seven days or more than seven days) over a two-week period. Prevalence ratios were estimated by Poisson regression.

**RESULTS:**

Evaluated by the PHQ-9 scale, 9.7% of the Brazilian adults had depression and 3.9% presented major depression. About 21.0% reported depressive mood and, in 34.9% of them, that feeling has been present for more than seven days. In individuals with major depression (PHQ-9), higher prevalence was found in almost all unhealthy behaviors analyzed, in particular, smoking (PR = 1.65), passive smoking (PR = 1.55), risk alcohol consumption (PR = 1.72), TV for ≥ 5 hours/day (PR = 2.13), consumption of fat meat (PR = 1.43) and soft drink (PR = 1.42). The prevalence ratios tended to be lower in those with minor depression. Similar results were observed in adults with depressive mood.

**CONCLUSIONS:**

This study detected relevant association between depression and health behaviors, in particular for smoking and physical activity. The associations found with the PHQ were similar to those observed with the application of a single question about depressive mood. Our results indicate the importance of assessing the presence of depression and the frequency and severity of symptoms when implementing actions for the promotion of healthy behaviors.

## RESUMO

**OBJETIVO:**

Avaliar a prevalência de comportamentos relacionados à saúde segundo a presença e tipo de depressão em adultos brasileiros.

**MÉTODOS:**

Com base em amostra de 49.025 adultos (18 a 59 anos) da Pesquisa Nacional de Saúde 2013, foram estimadas as prevalências de comportamentos relacionados à saúde (tabagismo, fumo passivo, consumo frequente ou de risco de álcool, atividade física de lazer, horas de TV e indicadores de padrão alimentar), segundo a presença de depressão (menor e maior), avaliada pela escala PHQ-9, e o relato de humor depressivo (em até sete dias ou em mais de sete dias) em um período de duas semanas. Razões de prevalências foram estimadas por meio de regressão de Poisson.

**RESULTADOS:**

Avaliados pela escala PHQ-9, 9,7% dos adultos brasileiros apresentaram depressão, e 3,9%, depressão maior. Cerca de 21,0% relataram humor depressivo e em 34,9% deles esse sentimento esteve presente por mais de sete dias. Nos indivíduos com depressão maior (PHQ-9), foram constatadas prevalências mais elevadas de quase todos os comportamentos não saudáveis analisados, principalmente tabagismo (RP = 1,65), fumo passivo (RP = 1,55), consumo de risco de álcool (RP = 1,72), horas de TV ≥ 5 h/dia (RP = 2,13), consumo de carne gordurosa (RP = 1,43) e refrigerante (RP = 1,42). As razões de prevalência tenderam a ser menores na depressão menor. Resultados similares foram observados nos adultos com humor depressivo.

**CONCLUSÕES:**

O estudo detectou relevante associação entre depressão e comportamentos de saúde, em especial, para tabagismo e atividade física. As associações encontradas com o uso do PHQ foram similares às observadas com a aplicação de uma única pergunta sobre humor depressivo. Os resultados do estudo sinalizam a importância da avaliação da presença de depressão e da frequência e gravidade dos sintomas quando da implementação de ações para a promoção de comportamentos saudáveis.

## INTRODUCTION

In the current epidemiological scenario, the high morbidity and mortality resulting from chronic noncommunicable diseases (CNCD) makes clear the importance of health-related behaviors, which are recognized risk factors for these diseases. This recognition has led national^a^ and international^b^ agencies to develop plans and strategies for controlling CNCDs, based on proposals for changing harmful behaviors, taking into account that health behaviors are socially determined^1^.

We must also highlight that depressive and other mental disorders, with strong presence in the epidemiological panorama, can interact, aggravate, or even constitute independent risk factors for chronic diseases^2^, in addition to greatly affect the adoption and maintenance of several health-related behaviors^3,4^.

Mental disorders, that reach 350 million people worldwide^c^, accounts for 7.4% of the disability-adjusted life years (DALY), 40.5% of which result from depressive disorders, and are also responsible for 22.9% of the years lived with disability (YLD)^5^. Mild, moderate, or severe depressive disorders, with or without psychotic symptoms, are characterized by the presence of depressive mood, loss of interest and pleasure, lack of energy, feelings of guilt or low self-esteem, sleep or appetite disorders, and low concentration^6^.

Evidences show that the presence of depression increases the risk of several cardiovascular diseases, including myocardial infarction, hemorrhagic stroke, and peripheral artery disease, and it can be considered an independent factor just as important as the classic risk factors for chronic diseases^7^. In the reverse direction, depression can arise as a result of disabilities and limitations that come with chronic diseases, drawing a vicious circle between depressive feelings and physical comorbidities^8^.

Regarding health behaviors, the association with depressive conditions has been evidenced for physical activity and sedentary lifestyle^4,9^, risky consumption of alcohol^10^, smoking^11,12^, and eating habits^13^.

Considering the importance of health behaviors in determining the pattern of health and morbidity/mortality of the population, as well as the high prevalence of mental and depressive disorders and their possible influence on adopting and changing behaviors harmful to health, this study aimed to evaluate the prevalence of several health behaviors according to the presence of depression and in different subgroups of depressed individuals.

## METHODS

This is a population-based cross-sectional study, using the database of the most complete health survey conducted in Brazil, the National Survey on Health (PNS), conducted by the Brazilian Institute of Geography and Statistics (IBGE) in partnership with the Brazilian Ministry of Health. Data collection of PNS was conducted in 2013 and 2014, in a probabilistic sample taken in three stages: in the first, the census tracts constituted the primary sampling units; in the second, households were randomly selected; and in the third, an individual aged 18 years or older living in those households was randomly selected. At each stage, the selection was carried out by simple random sampling.

PNS used three questionnaires: one for household characteristics, another on the household residents, and the third with information from the resident selected to participate in the survey. Details of the sampling plan and of other methodological aspects were published elsewhere^14^.

A total of 69,954 households covered by PNS 2013 were occupied, and 60,202 people aged 18 years or older were interviewed, resulting in a response rate of 86.1%. This study analyzed the information of the 49,025 individuals aged between 18 and 59 years.

The identification of depression was made with the Patient Health Questionnaire – 9 (PHQ-9), consisting of nine questions to evaluate the frequency of depressive symptoms in the last two weeks. The instrument, already validated in Brazil^6^, allows identifying individuals with high risk of major depression. People with five or more symptoms, frequent in more than seven days, were classified with major depression, and one of the symptoms should be “depressive mood” or “lack of interest or pleasure.” Those who showed less than two symptoms or negative response for “depressive mood” and “lack of interest or pleasure” were considered without depression. The remaining individuals were classified with minor depression. The simple report of the presence of depressive mood was also analyzed, from a single question of PHQ-9 (presence or absence of depression feeling in the two weeks before the interview), and those who had the symptom in up to seven days or more than seven days were discriminated.

For individuals identified by the PHQ-9 as having depression, the presence of a diagnosis of depression made by health professional was also examined.

The health behaviors selected for analysis were: current smoker (yes, no); smoking cessation (individuals who have stopped smoking among those who quit smoking or who continue smoking); passive smoker at home; usual alcohol intake once or more per month (yes, no); alcohol intake three or more times per week; risky alcohol consumption (heavy episodic drinking) estimated among non-abstainers, being considered the intake of four or more doses of drinks for women and five or more for men on a single occasion in the past 30 days, a dose being considered equivalent to a can of beer, a glass of wine, or a dose of distilled drink; leisure time physical activity (LTPA), being active individuals who reach the practice of at least 150 minutes per week of mild or moderate LTPA, or 75 minutes of vigorous LTPA, in free time, and inactive or insufficiently active those who do not; sedentary lifestyle (daily hours watching television: < 5, ≥ 5); weekly frequency of consumption of: uncooked vegetables, fruits, sweet foods (pieces of cake or pie, sweetmeat, chocolate, candy, cookies, or sweet biscuits), soft drinks or artificial juices (< 5 times, ≥ 5); fish consumption: never or less than once a week (yes, no); consumption of meet with excess fat (yes, no). Sex, age, and education level were used as control variables.

The prevalence, prevalence ratios (PR), and 95% confidence intervals (95%CI) of health behaviors in the different segments of individuals with and without depression were estimated. Differences were tested by Chi-square test. Prevalence ratios were estimated by Poisson regression with adjustment for sex, age, and education level. The analyses were carried out using the statistical software Stata 14.0, considering the effect of sample design, the nonresponse rates, and post-stratification weights.

PNS was approved by the National Research Ethics Committee (Process: 328,159, June 26, 2013). All participants signed the informed consent form.

## RESULTS

We analyzed data of 49,025 individuals with an average age of 37.0 years (95%CI 36.8–37.2), with 47.9% males. Of the Brazilian adults, 9.7% (95%CI 9.2–10.2) presented some degree of depression, identified by PHQ-9, and 3.9% (95%CI 3.6–4.2) had major depression. Among individuals with depression (major or minor), 27.6% (95%CI 25.2–29.3) reported having received, at some point in life, a clinical diagnosis of depression. Among Brazilian adults, 21.0% have felt depressed at some time in the two-week period (95%CI 20.3–21.7) and, in 34.9% of them, the depressive mood had persisted for more than seven days. Of the total of Brazilian adults, 7.2% (95%CI 6.8–7.7) had received a clinical diagnosis of depression at some point in life.

The prevalence of all indicators of harmful behaviors studied (except intake of alcohol three or more times a week, and low consumption of fruits) was higher in individuals identified by PHQ-9 with major depression. The prevalence ratios tended to be lower in those evaluated as having minor depression, although significant differences between major and minor depression have been detected only for risky alcohol intake and hours of TV (Table 1).

**Table 1:** Prevalence and prevalence ratio of health behavior indicators according to presence and type of depression (PHQ-9). PNS, 2013.

Health behavior	No depression (1)^a^ n = 43,881	With minor depression (2) n = 3,107	With major depression (3) n = 2,037	Adjusted PR^b^ 95%CI					

				(2/1)		(3/1)		(3/2)	
Smoking									
Current smoker	14.5	20.2	22.8	**1.52**	**1.32–1.75**	**1.65**	**1.38–1.97**	1.01	0.97–1.06
Stopped smoking	49.2	44.2	48.8	**0.78**	**0.65–0.94**	0.93	0.74–1.17	1.03	0.97–1.08
Passive smoker at home	10.3	15.8	16.7	**1.48**	**1.22–1.79**	**1.55**	**1.25–1.95**	1.00	0.95–1.06
Alcohol intake									
Regular intake (≥ 1 time/month)	29.6	22.8	22.0	0.96	0.83–1.10	1.01	0.83–1.22	1.00	0.97–1.04
Intake ≥ 3 times/week	6.0	6.6	5.6	**1.55**	**1.18–2.04**	1.41	0.99–2.01	0.98	0.90–1.06
Risky intake among drinkers	35.5	35.4	43.0	1.17	0.95–1.44	**1.72**	**1.34–2.21**	**1.07**	**1.02–1.13**
Physical activity/sedentary lifestyle									
Inactive or insufficiently active at leisure time	74.7	82.2	86.9	**1.25**	**1.06–1.48**	**1.57**	**1.28–1.94**	1.04	1.00–1.09
TV for ≥ 5 hours	11.6	18.9	24.5	**1.58**	**1.36–1.84**	**2.13**	**1.79–2.5**	**1.06**	**1.02–1.10**
Feeding									
Uncooked vegetables consumption ≤ 5 times/week	54.1	55.1	60.3	1.08	0.95–1.22	**1.30**	**1.12–1.50**	1.04	1.00–1.07
Fruit consumption ≤ 5 times/week	61.6	61.3	61.2	1.06	0.94–1.19	1.02	0.88–1.19	1.00	0.97–1.03
Fatty meat consumption	38.8	40.9	43.8	**1.26**	**1.12–1.41**	**1.43**	**1.23–1.66**	1.03	0.99–1.06
No fish consumption in the week	45.5	53.4	53.7	**1.32**	**1.18–1.48**	**1.32**	**1.14–1.53**	1.00	0.99–1.03
Sweet food consumption ≥ 5 times in the week	22.1	29.3	26.8	**1.17**	**1.02–1.33**	**1.20**	**1.01–1.42**	0.99	0.95–1.03
Consumption of soft drink or artificial juice ≥ 5 times in the week	25.8	26.8	26.2	**1.49**	**1.30–1.70**	**1.42**	**1.20–1.69**	1.01	0.97–1.04

Statistically significant values are presented in bold.

^a^ Reference category.

^b^ Adjusted by age, sex, and education level.

Considering only the report of depressive mood, obtained from a single question of PHQ-9, all the harmful behaviors studied (except low consumption of fruit) were more prevalent in individuals who reported depressive mood lasting more than seven days. The prevalence ratios were significantly lower in those who presented depressive mood in up to seven days (Table 2).

**Table 2:** Prevalence and prevalence ratio of health behavior indicators according to presence and frequency of depressive feeling. PNS, 2013.

Health behavior	No report of depressive feeling (depressive mood) (1)^a^ n = 38,122	With report of depressive feeling in up to seven days in the two weeks prior (2) n = 6,987	With report of depressive feeling in more than seven days in the two weeks prior (3) n = 3,916	Adjusted PR^b^ 95%CI					

				(2/1)		(3/1)		(3/2)	
Smoking									
Current smoker	14.2	16.3	23.0	**1.20**	**1.09–1.33**	**1.67**	**1.48–1.88**	**1.06**	**1.03–1.09**
Stopped smoking	48.7	51.6	44.9	1.05	0.92–1.20	**0.82**	**0.70–0.97**	**0.96**	**0.92–0.99**
Passive smoker at home	9.9	13.9	15.7	**1.32**	**1.18–1.48**	**1.47**	**1.25–1.75**	1.02	0.98–1.06
Alcohol intake									
Regular intake (≥ 1 time/month)	30.0	26.5	22.4	1.09	1.00–1.96	1.00	0.88–1.14	0.99	0.96–1.02
Intake ≥ 3 times/week	6.1	5.8	5.5	**1.24**	**1.05–1.46**	**1.32**	**1.04–1.68**	1.01	0.96–1.06
Risky intake among drinkers	36.0	32.5	39.9	0.99	0.88–1.23	**1.44**	**1.19–1.74**	**1.07**	**1.03–1.11**
Physical activity/sedentary lifestyle									
Inactive or insufficiently active at leisure time	74.3	79.0	83.3	1.08	0.98–1.19	**1.20**	**1.03–1.40**	1.02	0.99–1.05
TV for ≥ 5 hours/day	11.6	13.1	21.3	1.08	0.97–1.22	**1.72**	**1.51–1.95**	**1.10**	**1.07–1.13**
Feeding									
Uncooked vegetables consumption ≤ 5 times/week	54.3	53.3	57.6	1.00	0.92–1.08	**1.15**	**1.03–1.28**	**1.03**	**1.00–1.06**
Fruit consumption ≤ 5 times/week	61.5	61.8	61.8	**1.08**	**1.00–1.16**	1.06	0.96–1.18	1.00	0.98–1.02
Fatty meat consumption	38.8	39.0	42.8	**1.13**	**1.04–1.24**	**1.35**	**1.21–1.50**	**1.03**	**1.01–1.06**
No fish consumption in the week	45.0	49.9	53.4	**1.17**	**1.08–1.27**	**1.32**	**1.19–1.46**	**1.02**	**1.00–1.04**
Sweet food consumption ≥ 5 times in the week	22.0	24.6	26.3	**1.11**	**1.02–1.21**	**1.16**	**1.03–1.29**	1.01	0.98–1.03
Consumption of soft drink or artificial juice ≥ 5 times in the week	25.8	26.2	25.8	**1.16**	**1.06–1.26**	**1.36**	**1.21–1.52**	**1.03**	**1.00–1.06**

Statistically significant values are presented in bold.

^a^ Reference category.

^b^ Adjusted by age, sex, and education level.

## DISCUSSION

Individuals with depression showed higher prevalence for almost all harmful behaviors studied and, in the segments with major depression, the prevalence ratios tended to be even higher. However, most of the differences observed between major and minor depression had no statistical significance. With the simple report of the presence of depressive mood, we identified a segment in which almost all harmful behaviors were more present. This presence was significantly higher in those who presented depressive mood for more than seven days in the two-week period.

The prevalence of 9.7% depression, 3.9% major depression, and 21.0% depressive mood in Brazilian adults, found in this study, are within the wide range reported in the literature, with studies using different methods for detecting depressive disorders. Data from European countries indicate that 6.7% of adults suffer from major depression and that 26.7% are affected by any kind of depression and anxiety^d^. In the United States, the prevalence of depressive disorder ranges from 9.0% to 23.5% among the American states and territories^15^. In a study conducted in the metropolitan region of São Paulo, the prevalence of major depression was estimated in 9.4%^16^. Another research developed in Brazil detected a prevalence of major depression of 8.2% for the city of São Paulo and of 6.0% for the city of Rio de Janeiro^17^.

The prevalence of the health behaviors examined in this research are close to the levels already identified by Vigitel for residents of the Brazilian state capitals^18^.

Smoking and sedentary lifestyle were the behaviors that showed the strongest associations with depressive symptoms. Smoking was 52% higher in individuals with minor depression, 65% higher in those with major depression, and 67% higher in the segment with report of depressive mood lasting more than seven days. Cross-sectional studies have found higher prevalence of depressive symptoms in smokers^10^. Follow-up studies indicate that smoking can be a predictor of depression^19^ and that, on the other hand, depression can lead to increased risk of smoking initiation^12^. A cohort research with follow-up of 25 years found a probability of suicidal ideation 3.4 times higher in people who smoked 20 or more cigarettes per day, when compared to nonsmokers^20^.

Regarding smoking cessation, researches have also found relationship with mental health^21,22^. According to the results of a study with 3,403 adults, the higher depression scores were associated with the lower smoking cessation^21^. Reviewing intervention researches, Meer et al. concluded that the control of humor combined with standard treatments is an important component to increase the percentage of cessation success^22^. The association of passive smoking at home with presence of depression, identified in this study, has also been found by Jung et al.^23^ for the female population in a study held in Korea.

Harmful alcohol consumption is associated with several chronic diseases and accidents and violence, resulting in severe social, economic, and legal commitments, which led the World Health Organization to propose to its member countries a 10% reduction of the risky alcohol consumption until 2025^d^. In addition to the mentioned consequences, the risky alcohol consumption is significantly associated with depression and with the simple report of depressive mood, as identified in this study. Usual alcohol intake did not differ among those who reported depression or not, but the risky consumption was 72% higher among individuals with major depression and 44% higher in individuals with depressive mood for more than seven days.

Many studies have examined the association between harmful use of alcohol and different types of depressive disorders, but the results do not always converge to a clear pattern of association. In a review study, Pedrelli et al.^24^ found that researches indicate increased risk of depressive symptoms and major depression in adolescents who consume alcohol frequently and are heavy drinkers and suggest that the causal relationship between alcohol and depression is bidirectional. Jetelina et al.^25^ have found, in Central America, an alcohol addiction four times higher in individuals with major depressive disorder. Study developed in the United States with middle-aged and older adults found that individuals with depression showed a probability 18% (6% to 31%) higher of becoming heavy drinkers^3^. Boden and Fergusson^10^, in literature review, suggest a plausible causal association between harmful use of alcohol and emergence of major depressive disorder, with more frequency than the association in the reverse direction. Most research notes the association between alcohol and depression, and a genetic basis was identified for this occurrence. In a meta-analysis, Oo et al.^26^ confirm that individuals with homozygous S allele of the 5HTTLPR polymorphism have increased risk of major depression and alcohol addiction.

Regarding leisure time physical inactivity and sedentary lifestyle, in this study, individuals with depressive symptoms were more inactive and sedentary, and the prevalence was higher in individuals with major depression. The association between physical inactivity at leisure and depressive feelings has been observed in the literature^4^, and there is evidence that physical activity may be a significant factor for the promotion of mental health and well-being^27^. According to Strohle’s systematic review, cross-sectional studies show greater association of physical activity with lower prevalence of depression, and follow-up researches indicate a protective effect of physical exercise on the risk of developing depressive disorders^4^. A randomized and controlled clinical trial analyzing doses of antidepressant treatments combined with aerobic exercises found that the group that participated in the exercise program started to require smaller doses of medication^28^.

Sedentary behavior, evaluated by the indicator “time watching TV,” has stood out for its association with worse indices of physical health and higher rates of mortality^29^. However, studies evaluating possible mental health damage induced by physical inactivity are still scarce. A study conducted with data from the Scottish Health Survey (SHS) points out that the longest time watching TV is associated with worse physical and mental health indices in adults^9^.

Regarding the association between depression and feeding, all indicators of unhealthy feeding evaluated in this study were more prevalent in individuals with minor or major depression (PHQ-9) and with report of depressive mood lasting more than seven days.

Many studies evaluate the relationship between feeding and cardiovascular diseases, cancer, and metabolic diseases; however, the association between eating patterns and mental health is still an emerging topic in the field of nutritional epidemiology^30^.

Some studies have pointed an association between eating patterns and depression. A longitudinal study conducted in Australia found less incidence of depressive symptoms in women who adopted the Mediterranean diet^13^. Another Australian study developed with men and women aged between 50 and 69 years also found inverse association between Mediterranean diet and presence of psychological distress (OR = 0.72)^31^. A research conducted with middle-aged Finnish men identified that followers of the “prudent” eating pattern, which includes fresh food, fish, and lean cheeses, presented 25% lower prevalence of major depression and that individuals with “Western” eating pattern, which includes sausages, fatty cheeses, and soft drinks, had a higher prevalence of these disorders (OR = 1.41)^32^.

Much of the evidence linking diet and depression comes from researches with cross-sectional design, which prevents to assess causal relationship, and few longitudinal studies evaluated this association. One of them accompanied more than 10 thousand participants for six years and found association between consumption of trans fats, fast food, and bakery products with higher risk of depression^33^. A study of systematic review identified as factors associated with lower risk of depression the consumption of folate, omega-3, and monounsaturated fatty acids, from foods such as olive oil, fish, fruits, vegetables, nuts, and legumes. A research developed in China identified lower probability of depressive symptoms in individuals who consumed fish at least three days in the week^34^.

The assessment of the results produced in this study needs to consider some limitations. The identification of major and minor depression was performed using the algorithm proposed for the PHQ-9 tool. Recent meta-analysis of studies that validated the used algorithm obtained 58% sensitivity and 94% specificity for the diagnosis of major depression^35^. Thus, it is likely that a proportion of people with major depression (about 40%) has been classified as having minor depression by PHQ-9. This may have influenced the results in exaggerating the association of risk behaviors with minor depression. Anyway, the results reveal consistent patterns and the presence of gradients in the expected direction. All the information about health behaviors can also undergo information bias. The broad dissemination of information about harmful behaviors to health can take part of respondents to sub-report practices recognized as unhealthy. It is worth noting, however, that the questionnaire used in PNS in the item on health behaviors is similar to that of Vigitel, and researches that used some of the generated indicators prove their validity^36^.

This is the first national representative study that evaluated the association between health risk behaviors and depression in Brazilian adults. Depression was assessed by a standardized and validated instrument, allowing comparisons with national and international studies. The results also indicate that, with a single question about depressive mood, it was possible to detect associations with similar magnitude and even verify that individuals with longer depressive mood showed prevalence significantly higher than those with shorter duration of the symptom.

This study shows an important association between behaviors considered as risk for health by the World Health Organization (smoking, alcohol consumption, inadequate nutrition, physical inactivity, and sedentary lifestyle) and presence of depression. Longitudinal studies have corroborated the bidirectional nature of these events, showing that behaviors harmful to physical health may also be involved in the occurrence of depressive conditions, and that the presence of these disorders can greatly affect the adoption of these behaviors. For some factors, such as smoking, risky alcohol consumption, physical inactivity and sedentary lifestyle, we observe strong evidence of this association in the literature, corroborating the results found in this study, whereas for indicators of inadequate diet this association is still unclear.

We highlight that our results, by identifying the association between health behaviors and depression, reinforce the relevance of actions for promoting healthy behaviors, both for the positive effect they exert regarding mental health and the control of other chronic noncommunicable diseases. On the other hand, it is important to consider issues related to the mental health of population segments when new healthy behaviors are desired and encouraged.

## Footnotes

a Brasil. Ministério da Saúde. Secretaria de Vigilância em Saúde. Departamento de Análise de Situação de Saúde. Plano de ações estratégicas para o enfrentamento das doenças crônicas não transmissíveis (DCNT) no Brasil 2011-2022. Brasília: Ministério da Saúde; 2011.

b Word Health Organization – Global status report on non-communicable diseases, 2010. Geneva: WHO; 2011. Available from: http://www.who.int/nmh/publications/ncd_report_full_en.pdf.

c World Health Organization. Sixty-fifth world health assembly. Geneva: WHO; 2012. Available from: http://www.who.int/mediacentre/events/2012/wha65/journal/en/index4.html

d Word Health Organization – Global status report on alcohol and health 2014. Geneva: WHO; 2014. Available from: http://www.who.int/substance_abuse/publications/global_alcohol_report/en/

## References

[1] Jarvis M, Wardle J, Marmot M, Wilkinson RG (1999). Social patterning of individual health behaviours: the case of cigarette smoking. Social determinants of health.

[2] Dhar AK, Barton DA (2016). Depression and the link with cardiovascular disease. Front Psychiatry.

[3] An R, Xiang X (2015). Smoking, heavy drinking, and depression among U.S. middle-aged and older adults. Prev Med.

[4] Ströhle A (2009). Physical activity, exercise, depression and anxiety disorders. J Neural Transm (Vienna).

[5] Whiteford HA, Degenhardt L, Rehm J, Baxter AJ, Ferrari AJ, Erskine HE (2013). Global burden of disease attributable to mental and substance use disorders: findings from the Global Burden of Disease Study 2010. Lancet.

[6] Santos IS, Tavares BF, Munhoz TN, Almeida LSP, Silva NTB, Tams BD (2013). Sensibilidade e especificidade do Patient Health Questionnaire-9 (PHQ-9) entre adultos da população geral. Cad Saude Publica.

[7] Daskalopoulou M, George J, Walters K, Osborn DP, Batty GD, Stogiannis D (2016). Depression as a risk factor for the initial presentation of twelve cardiac, cerebrovascular, and peripheral arterial diseases: Data Linkage Study of 1.9 Million Women and Men. PLoS One.

[8] Katon W, Lin EH, Kroenke K (2007). The association of depression and anxiety with medical symptom burden in patients with chronic medical illness. Gen Hosp Psychiatry.

[9] Hamer M, Stamatakis E, Mishra GD (2010). Television- and screen-based activity and mental well-being in adults. Am J Prev Med.

[10] Boden JM, Fergusson DM (2011). Alcohol and depression. Addiction.

[11] Gigantesco A, Ferrante G, Baldissera S, Masocco M, PASSI coordinating group (2015). Depressive symptoms and behavior-related risk factors, Italian Population-Based Surveillance System, 2013. Prev Chronic Dis.

[12] Patton GC, Carlin JB, Coffey C, Wolfe R, Hibbert M, Bowes G (1988). Depression, anxiety, and smoking initiation: a prospective study over 3 years. Am J Public Health.

[13] Rienks J, Dobson AJ, Mishra GD (2013). Mediterranean dietary pattern and prevalence and incidence of depressive symptoms in mid-aged women: results from a large community-based prospective study. Eur J Clin Nutr.

[14] Szwarcwald CL, Malta DC, Pereira CA, Vieira MLFP, Conde WL, Souza PRB (2014). Pesquisa Nacional de Saúde no Brasil: concepção e metodologia de aplicação. Cienc Saude Coletiva.

[15] Chowdhury PP, Mawokomatanda T, Xu F, Gamble S, Flegel D, Pierannunzi C (2016). Surveillance for certain health behaviors, chronic diseases, and conditions, access to health care, and use of preventive health services among states and selected local areas - Behavioral Risk Factor Surveillance System, United States, 2012. MMWR Surveill Summ.

[16] Andrade LH, Wang YP, Andreoni S, Silveira CM, Alexandrino-Silva C, Siu ER (2012). Mental disorders in megacities: findings from the São Paulo Megacity Mental Health Survey, Brazil. PLoS One.

[17] Ribeiro WS, Mari JJ, Quintana MI, Dewey ME, Evans-Lacko S, Vilete LMP (2013). The impact of epidemic violence on the prevalence of psychiatric disorders in Sao Paulo and Rio de Janeiro, Brazil. PLoS One.

[18] Ministério da Saúde (BR), Secretaria de Vigilância em Saúde, Departamento de Vigilância de Doenças e Agravos não Transmissíveis e Promoção da Saúde (2015). VIGITEL Brasil 2014: vigilância de fatores de risco e proteção para doenças crônicas por inquérito telefônico.

[19] Green BH, Copeland JR, Dewey ME, Sharma V, Saunders PA, Davidson IA (1992). Risk factors for depression in elderly people: a prospective study. Acta Psychiatr Scand.

[20] Boden JM, Ferguson, Horwood LJ (2008). Cigarette smoking and suicidal behavior: results from a 25-year longitudinal study. Psychol Med.

[21] Haukkala A, Uutela A, Vartiainen E, McAlister A, Knekt P (2000). Depression and smoking cessation: the role of motivation and self-efficacy. Addict Behav.

[22] Meer RM, Willemsen MC, Smit F, Cuijpers P (2013). Smoking cessation interventions for smokers with current or past depression. Cochrane Database Syst Rev.

[23] Jung SJ, Shin A, Kang D (2015). Active smoking and exposure to secondhand smoke and their relationship to depressive symptoms in the Korea National Health and Nutrition Examination Survey (KNHANES). BMC Public Health.

[24] Pedrelli P, Shapero B, Archibald A, Dale C (2016). Alcohol use and depression during adolescence and young adulthood: a summary and interpretation of mixed findings. Curr Addict Rep.

[25] Jetelina KK, Reingle Gonzalez JM, Vaeth PA, Mills BA, Caetano R (2016). An investigation of the relationship between alcohol use and major depressive disorder across Hispanic national groups. Alcohol Clin Exp Res.

[26] Oo KZ, Aung YK, Jenkins MA, Win AK (2016). Associations of 5HTTLPR polymorphism with major depressive disorder and alcohol dependence: a systematic review and meta-analysis. Aust N Z J Psychiatry.

[27] Penedo FJ, Dahn JR (2005). Exercise and well-being: a review of mental and physical health benefits associated with physical activity. Curr Opin Psychiatry.

[28] Siqueira CC, Valiengo LL, Carvalho AF, Santos-Silva PR, Missio G, Sousa RT (2016). Antidepressant efficacy of adjunctive aerobic activity and associated biomarkers in major depression: a 4-week, randomized, single Blind, controlled clinical trial. PLoS One.

[29] Chau JY, Grunseit AC, Chey T, Stamatakis E, Brown WJ, Bauman AE (2013). Daily sitting time and all-cause mortality: a meta-analysis. PLoS One.

[30] Sarris J, Logan AC, Akbaraly TN, Amminger GP, Balanzá-Martínez V, Freeman MP (2015). Nutritional medicine as mainstream in psychiatry. Lancet Psychiatry.

[31] Hodge A, Almeida OP, English DR, Giles GG, Flicker L (2013). Patterns of dietary intake and psychological distress in older Australians: benefits not just from a Mediterranean diet. Int Psychogeriatr.

[32] Ruusunen A, Lehto SM, Mursu J, Tolmunen T, Tuomainen TP, Kauhanen J (2014). Dietary patterns are associated with the prevalence of elevated depressive symptoms and the risk of getting a hospital discharge diagnosis of depression in middle-aged or older Finnish men. J Affect Disord.

[33] Sánchez-Villegas A, Toledo E, Irala J, Ruiz-Canela M, Pla-Vidal J, Martínez-González MA (2012). Fast-food and commercial baked goods consumption and the risk of depression. Public Health Nutr.

[34] Wu D, Feng L, Gao Q, Li JL, Rajendran KS, Wong JC (2016). Association between fish intake and depressive symptoms among community-living older Chinese adults in Singapore: a cross-sectional study. J Nutr Health Aging.

[35] Manea L, Gilbody S, McMillan D (2015). A diagnostic meta-analysis of the Patient Health Questionnaire-9 (PHQ-9) algorithm scoring method as a screen for depression. Gen Hosp Psychiatry.

[36] Monteiro CA, Florindo AA, Claro RM, Moura EC (2008). Validade de indicadores de atividade física e sedentarismo obtidos por inquérito telefônico. Rev Saude Publica.

